# The Efficient Knot: Redefining Simplicity in High-Tension Wound Closure

**DOI:** 10.7759/cureus.101093

**Published:** 2026-01-08

**Authors:** Ioannis Kyriazidis, Leonidas Pavlidis, Athanasios Papas, Hieronymus P Stevens, Jacques Van Der Meulen

**Affiliations:** 1 Department of Plastic and Reconstructive Surgery, General Hospital Papageorgiou, Thessaloniki, GRC; 2 Department of Plastic and Reconstructive Surgery, School of Medicine, Faculty of Health Sciences, Aristotle University of Thessaloniki, Thessaloniki, GRC; 3 Plastic Surgery, Velthuis Clinic, Rotterdam, NLD

**Keywords:** dermatologic surgery, high tension wound, self-locking knot, single-handed suture, surgical knot, surgical technique, suture tutorial, suturing technique, wound approximation, wound closure

## Abstract

Closing wounds under high tension often requires complex maneuvers or significant non-dominant hand involvement. We present a novel, single-handed suturing technique that simplifies this process. Following standard loop formation, the needle holder passes under the long suture end, over the short end, and under again to grasp the free thread, creating a self-locking configuration. This technique limits the non-dominant hand to simply holding the suture end while maintaining precise tissue approximation. It works with both monofilament and braided sutures and is applicable to skin closure, fascial repair, and anatomically challenging locations. The straightforward execution makes it accessible to surgeons across all skill levels.

## Introduction

Closing wounds under high tension remains a common challenge in dermatologic and plastic surgery, with various techniques having been described in the literature. These range from simple pulley stitches to more complex methods like buried vertical mattress sutures and loop techniques [1-5], each attempting to bypass the traditional drawbacks of suturing under tension using needle holders, namely, the need for surgical assistance, additional epidermal punctures, and complex knot configurations that can be challenging to master. Furthermore, maintaining precise tissue approximation while securing the first knot can be technically demanding, particularly in anatomically challenging locations or when working with delicate tissues.

Existing techniques for high-tension closure can be broadly categorized into pulley-based methods, which require additional epidermal punctures, and sliding knot techniques, which allow approximation before locking. The latter category includes the granny sliding knot [1], the Loma Linda loop [5], the double-loop dermal suture [2], and various other methods requiring active non-dominant hand participation [3,4]. While effective, these methods typically demand that the non-dominant hand actively maintain loops, apply counter-tension, and execute precisely timed release maneuvers during knot formation.

The technique presented here differs mechanically from previously described methods in a fundamental way: rather than creating a sliding knot that locks upon approximation through loop manipulation, it employs a specific under-over-under needle holder trajectory that generates a self-locking configuration during a single fluid movement. This configuration traps friction between the suture strands without requiring the non-dominant hand to maintain loops or apply counter-tension during the locking phase.

We present a novel, simple, straightforward suturing technique that elegantly addresses these challenges through a fluid movement that can be performed single-handedly and is particularly valuable in anatomically challenging locations where previous techniques might be cumbersome or impractical.

## Technical report

For right-handed surgeons, the technique proceeds as follows:

Step 1: Create a standard single loop around the needle holder following initial suture placement through the tissue.

**Figure 1:**
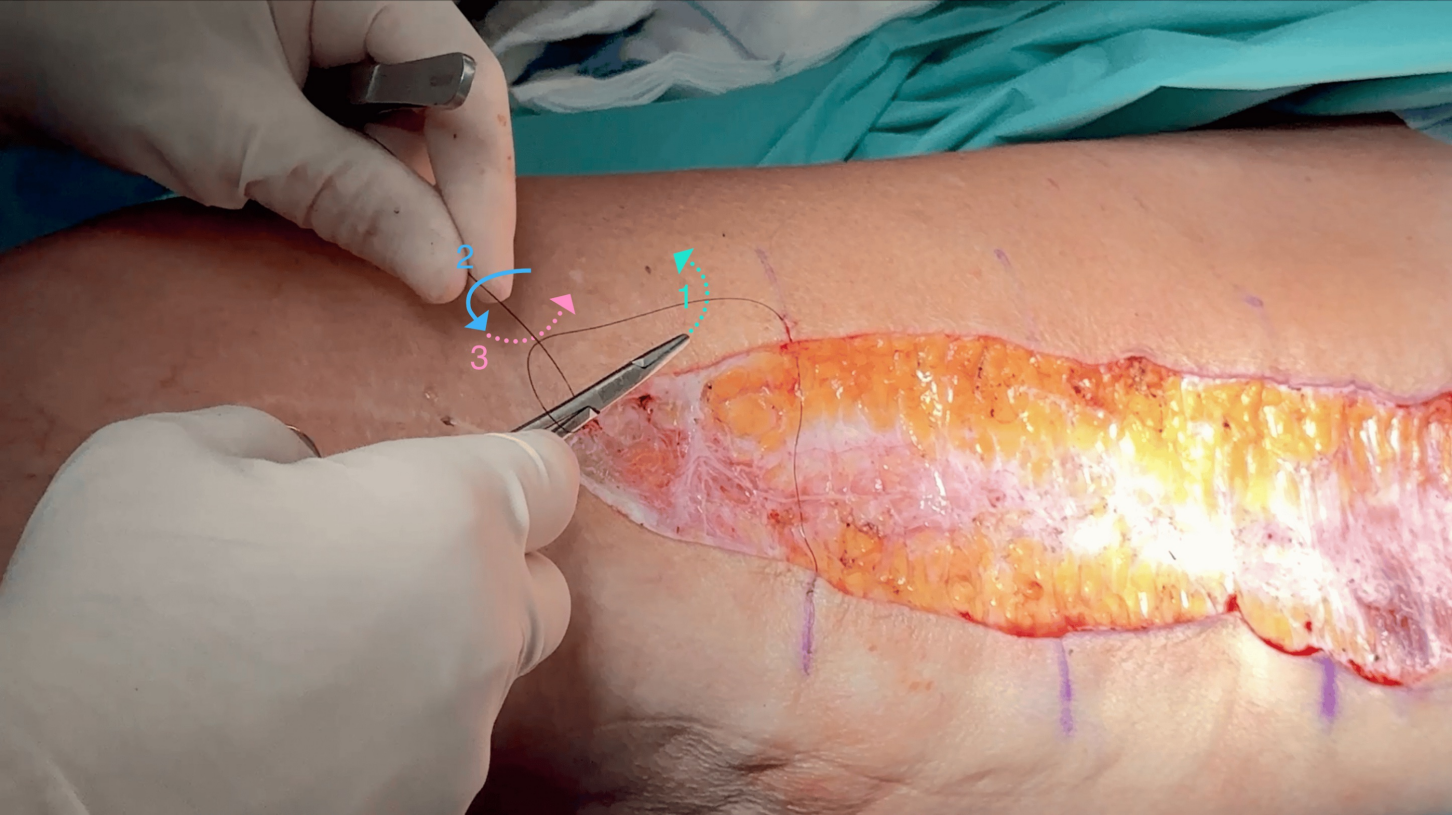
Intraoperative photograph demonstrating the Efficient Knot technique during high-tension wound closure Numbered arrows indicate the sequential needle holder trajectory: (1) passage under the long (needle-side) suture end; (2) transition over the short (tail) end; (3) passage under the short end again to grasp the free thread. Dotted lines represent passage of the needle holder under the suture strands; solid lines represent passage over. Note the non-dominant hand (upper left) holds only the suture tail in a stationary position throughout the maneuver—no active loop manipulation is required.

Step 2: Guide the needle holder tip under the long (needle-side) end of the suture (arrow 1 in Figure 1).

Step 3: Transition the needle holder over the short (tail) end of the suture (arrow 2 in Figure 1).

Step 4: Pass the needle holder under the short end again to grasp the free end of the thread (arrow 3 in Figure 1).

Step 5: After securing the thread, advance the knot with controlled tension while the non-dominant hand simply holds the suture tail in a stationary position.

Step 6: Complete the closure with a clockwise double surgeon's knot followed by a single counterclockwise throw for maximum security.

This specific under-over-under trajectory is critical to the technique's mechanism: it creates two points of friction lock between the suture strands while simultaneously allowing controlled advancement of the knot. The geometric configuration ensures that tension applied to the free end during knot advancement translates directly to wound edge approximation rather than premature knot locking, a common limitation encountered with traditional granny knot configurations and other sliding knot methods. The complete technique is also demonstrated in Video 1.

**Video 1:** The Efficient Knot This video demonstrates a novel suturing technique for closing wounds under high tension. The technique can be performed single-handedly and is particularly useful in anatomically challenging locations.

## Discussion

Our current approach builds upon previous work on efficient wound closure under high tension [1-5], but offers distinct advantages in simplicity and efficiency (Table 1). The key distinction lies in the role of the non-dominant hand: while established techniques such as the Loma Linda loop [5] and the double-loop dermal suture [2] require active finger coordination during knot advancement, the present technique fundamentally simplifies this requirement.

**Table 1:** Comparison of high-tension wound closure techniques SALT: simultaneous approximation and locking technique

Technique	Non-dominant Hand Role	Mechanism	Complexity
Granny sliding knot [1]	Holds suture tail; requires precise sliding motion	Granny configuration slides along the suture	Moderate
Double-loop dermal [2]	The middle finger maintains tension and creates a second loop	Finger-assisted tension redistribution	Moderate-high
Loop capture technique [3]	Grasps loop between fingers; timed release	Loop capture and release	Moderate
SALT knot [4]	Pushes the wound edge while holding the suture	Double granny slides with edge manipulation	Moderate
Loma Linda loop [5]	Pinches loop during advancement; timed release	Loop release after approximation	Moderate
Efficient Knot (present)	Holds suture tail only (passive/stationary)	Self-locking via under-over-under trajectory	Low

The technique described here offers several distinct advantages over existing methods. Most notably, it addresses a limitation common to all previously described approaches: the requirement for active non-dominant hand participation. Although Kyriazidis et al. [3] aptly titled their contribution, wound closure under tension "takes brains, not brawn", yet even that technique requires the non-dominant hand to actively grasp and release the loop at precise moments. The Efficient Knot takes this philosophy further by eliminating active non-dominant hand maneuvers entirely, limiting that hand to simply holding the suture end in a stationary position.

Self-locking mechanism

The under-over-under needle holder trajectory creates a geometric configuration that automatically generates friction between suture strands as tension is applied. Unlike methods that rely on the surgeon's fingers to maintain loop position until the critical moment of approximation [2,3,5], this technique's locking mechanism is inherent to the knot structure itself.

Anatomically challenging locations

The technique proves particularly valuable in deep or confined operative fields where complex left-hand maneuvers might be cumbersome. Previous techniques requiring active finger manipulation [2,3,5] can be difficult to execute when working through small incisions or in areas with limited access.

Versatility

The technique performs equally well with both monofilament and braided sutures and extends beyond simple skin closure to situations requiring controlled approximation of any tissue type, including deep dermal layers, fascia, and tendon repair.

Accessibility

The straightforward execution makes it accessible to surgeons at all skill levels. Unlike techniques requiring precisely timed loop release [3,5] or coordinated finger-instrument movements [2], the learning curve for the Efficient Knot is minimal.

Clinical experience

This technique has been employed by the authors in clinical practice across diverse applications, including skin closure following tumor excision, fascial repair, and wound closure in anatomically challenging locations. While systematic outcome data are not presented in this technical report, no instances of knot failure, suture breakage, or wound dehiscence attributable to the technique have been observed in our experience. Formal comparative studies examining loop security, failure rates, and learning curves would strengthen the evidence base for this approach.

Limitations

This report describes the technical aspects of the knot without comparative biomechanical testing or prospective clinical outcome data. The claims of superiority in simplicity are based on the authors' subjective assessment of the mechanical requirements of each technique rather than objective metrics. Future studies comparing loop security, knot failure rates, operative time, and learning curves against established techniques would provide stronger evidence to support the adoption of this method.

## Conclusions

This novel, single-handed suturing technique appears to provide an efficient and reproducible method for high-tension wound closure. By minimizing non-dominant hand involvement to a passive holding role and utilizing a self-locking mechanism via a specific under-over-under needle holder trajectory, it may offer practical advantages over existing methods discussed earlier, in terms of simplicity and ease of execution.

The technique's versatility across tissue types and suture materials, combined with its accessibility to surgeons at all skill levels, suggests its potential value as an addition to the surgical armamentarium for managing high-tension wounds.
